# Supplementary figures and images for: Robust T cell activation requires an eIF3-driven burst in T cell receptor translation
Source: eLife. 2021 Dec 31;10:e74272. doi: 10.7554/eLife.74272 (PMC8758144; doi:10.7554/eLife.74272)

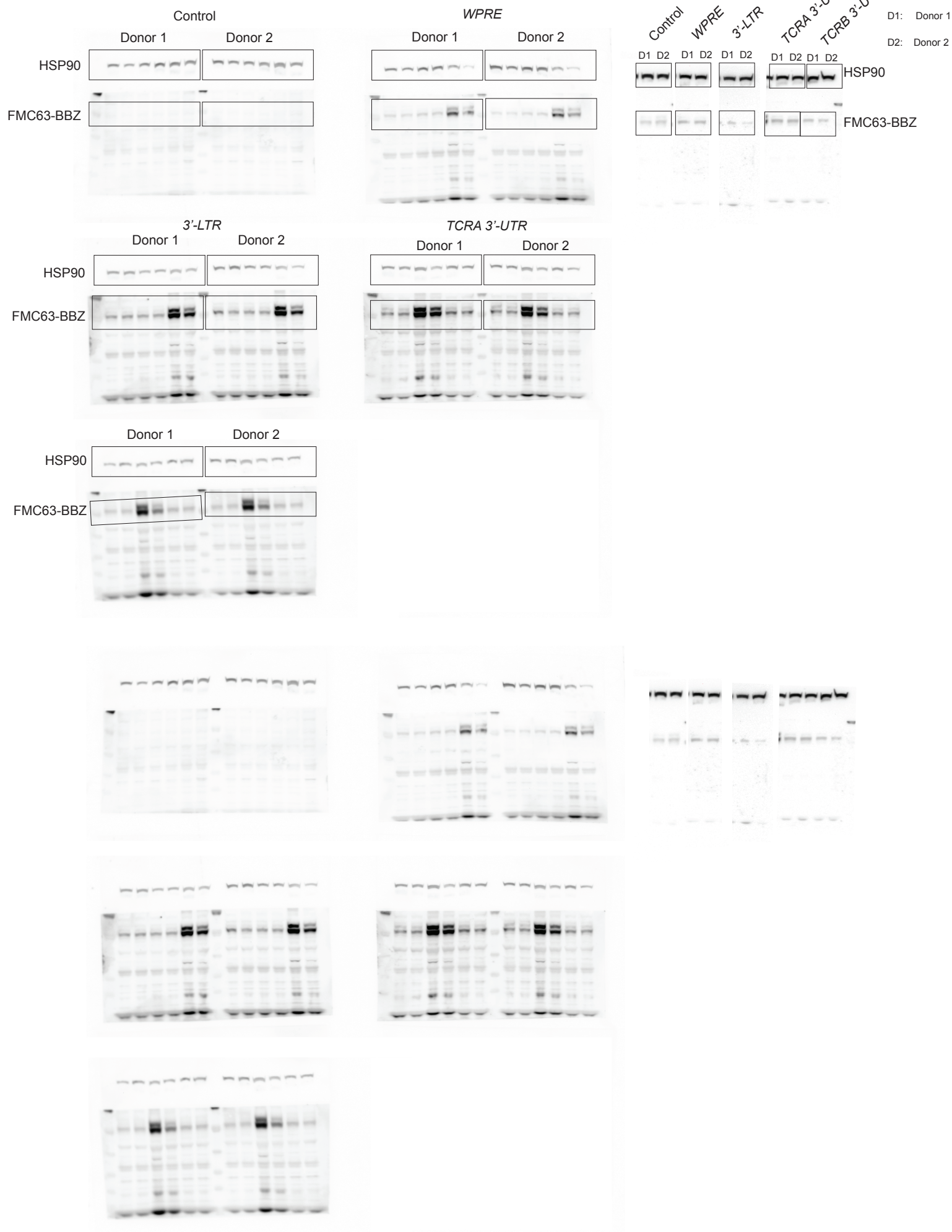

Supplement: Source data 1. — Boxed regions are the regions shown in the figures. [file elife-74272-supp7.zip › DeSilva_Source_Data_Gels-revised/Figure_6.pdf]

Figure 5 A

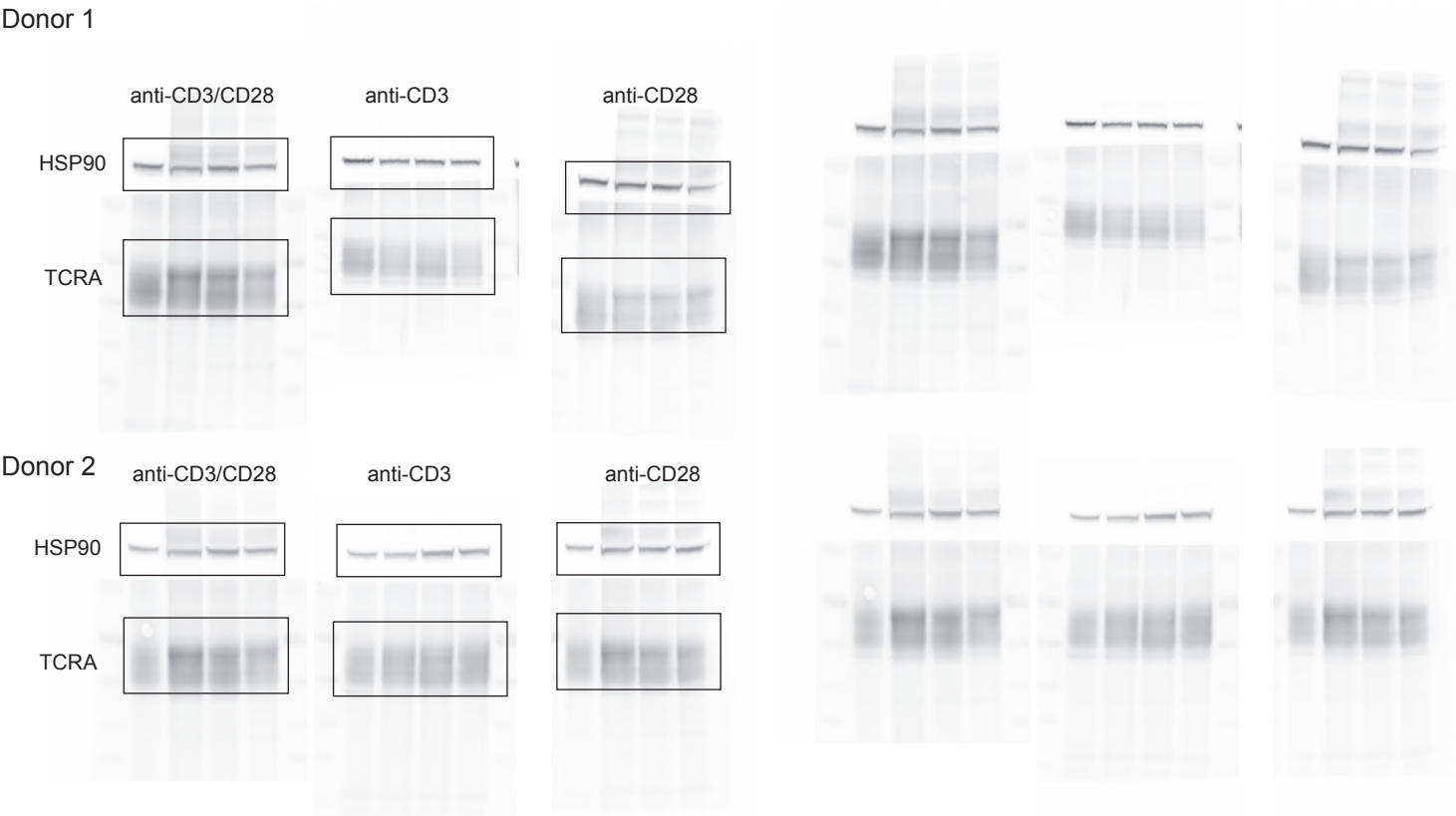

Figure 5 B

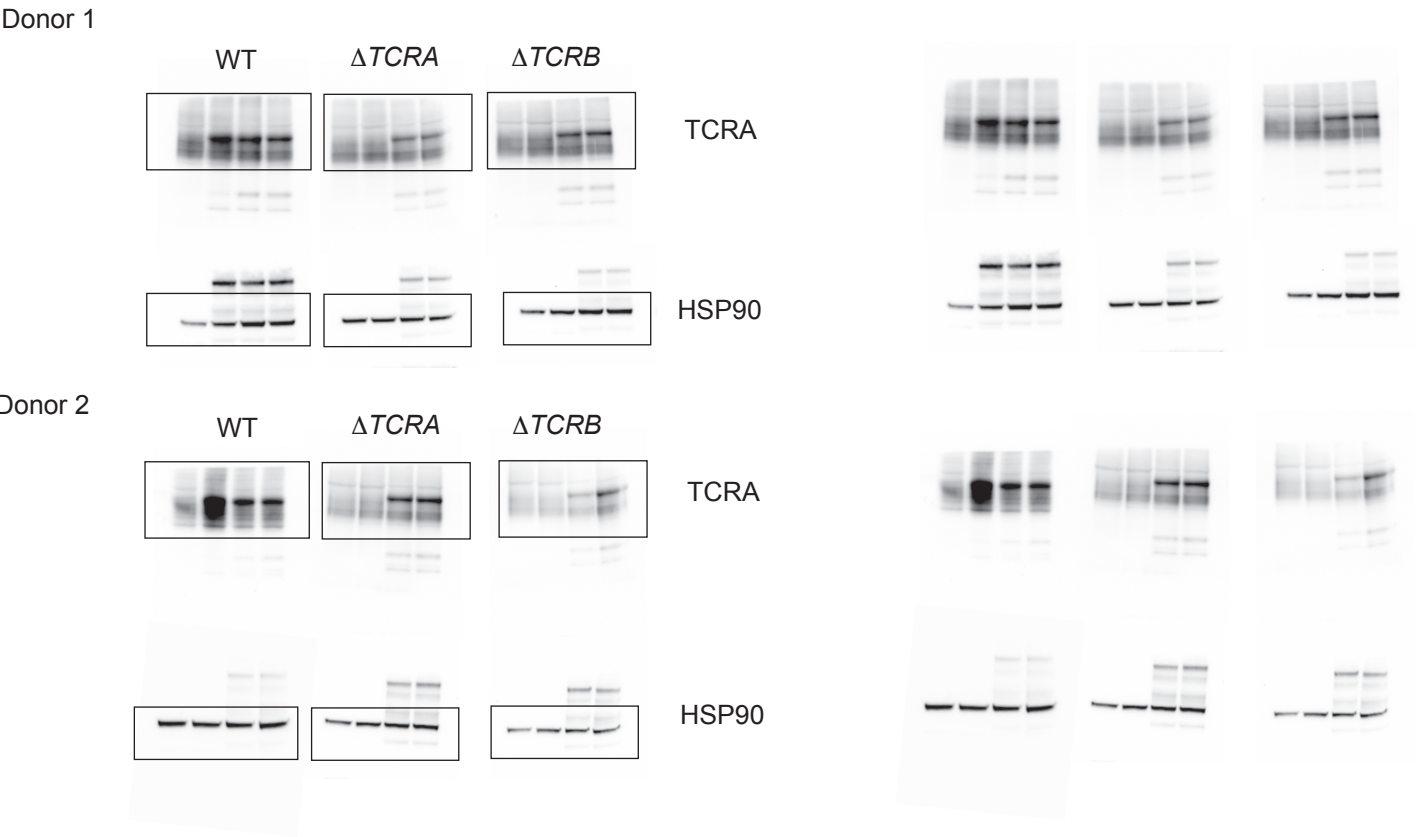

Supplement: Source data 1. — Boxed regions are the regions shown in the figures. [file elife-74272-supp7.zip › DeSilva_Source_Data_Gels-revised/Figure_5.pdf]

Figure\_4

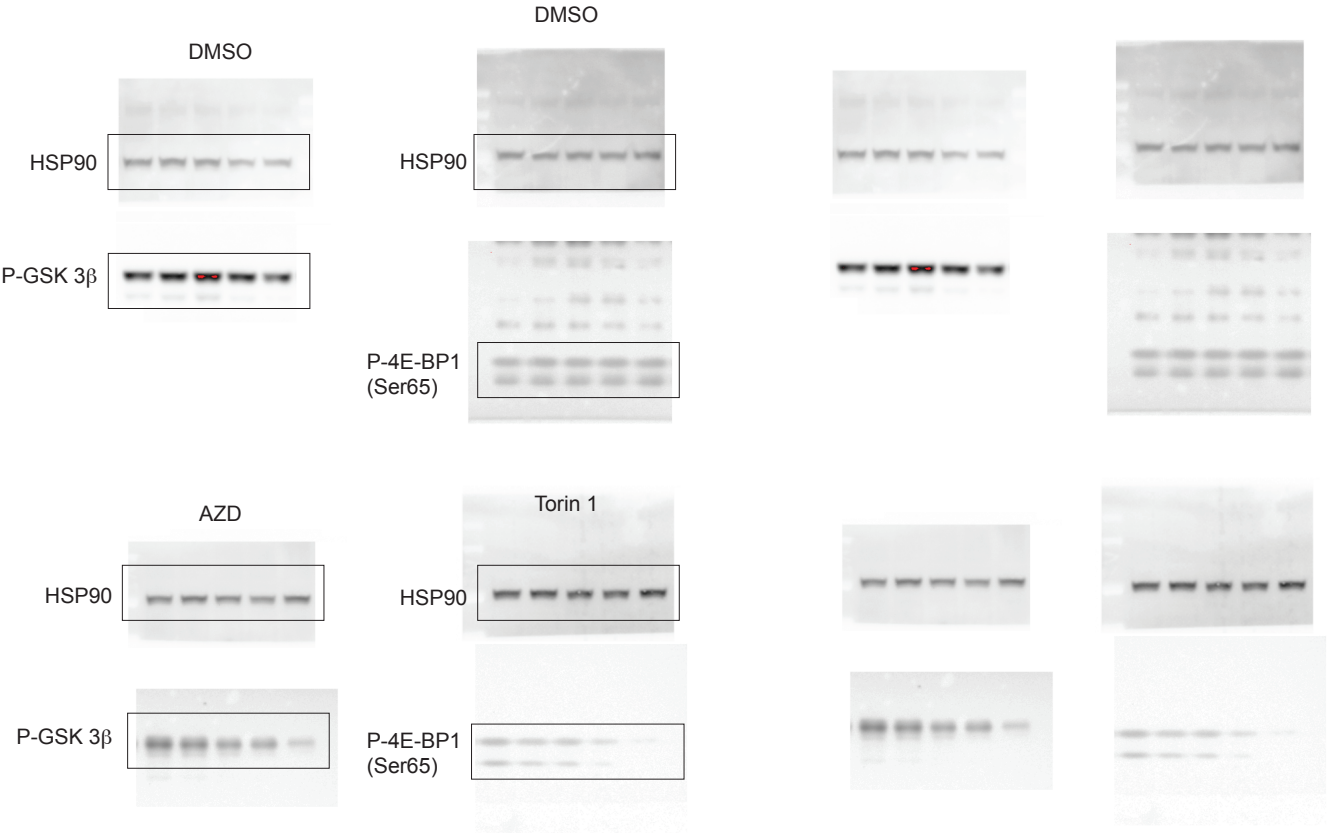

Supplement: Source data 1. — Boxed regions are the regions shown in the figures. [file elife-74272-supp7.zip › DeSilva_Source_Data_Gels-revised/Figure_4.pdf]

Figure 1B

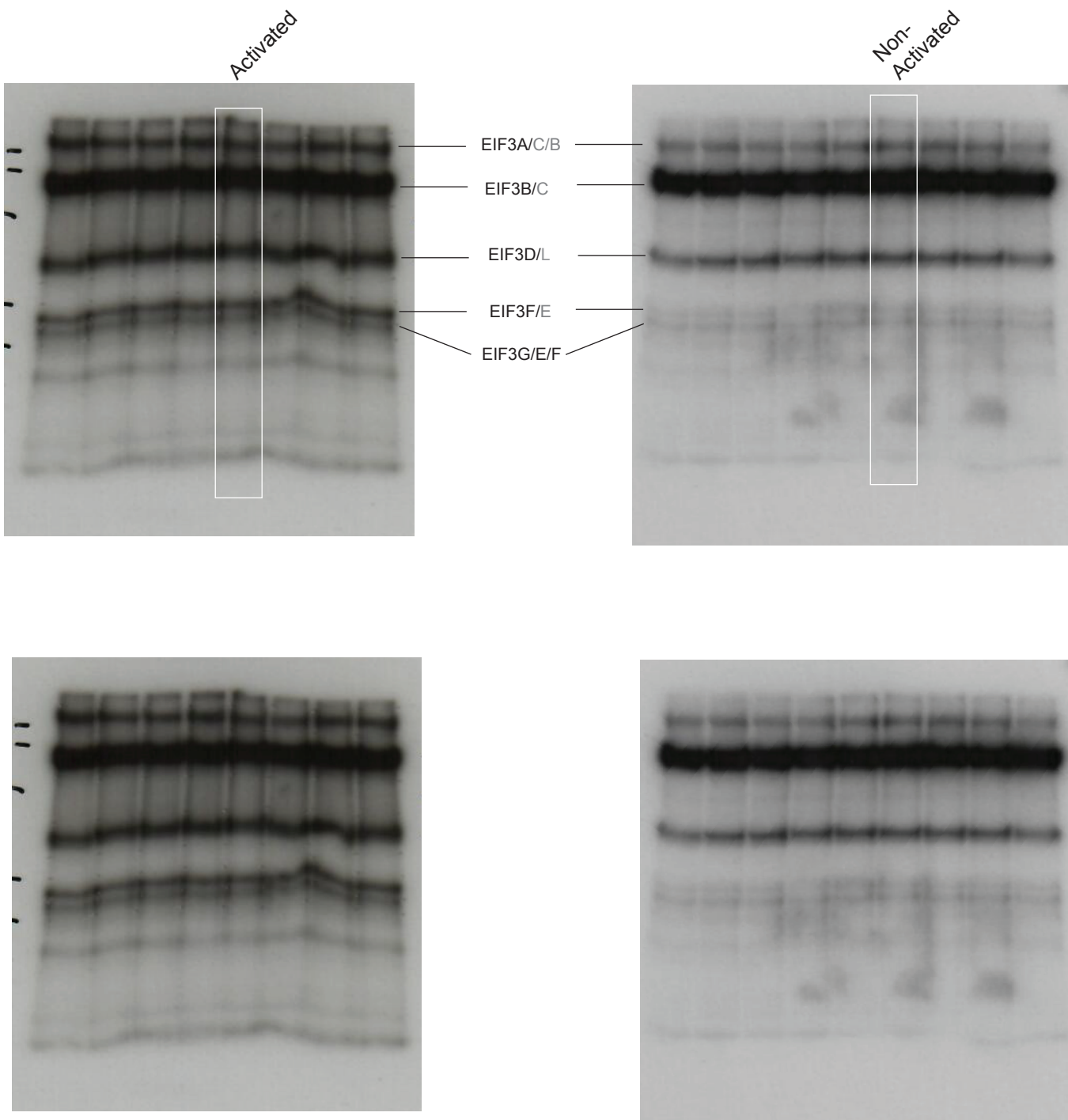

Supplement: Source data 1. — Boxed regions are the regions shown in the figures. [file elife-74272-supp7.zip › DeSilva_Source_Data_Gels-revised/Figure_1.pdf]

Figure\_1\_S1A

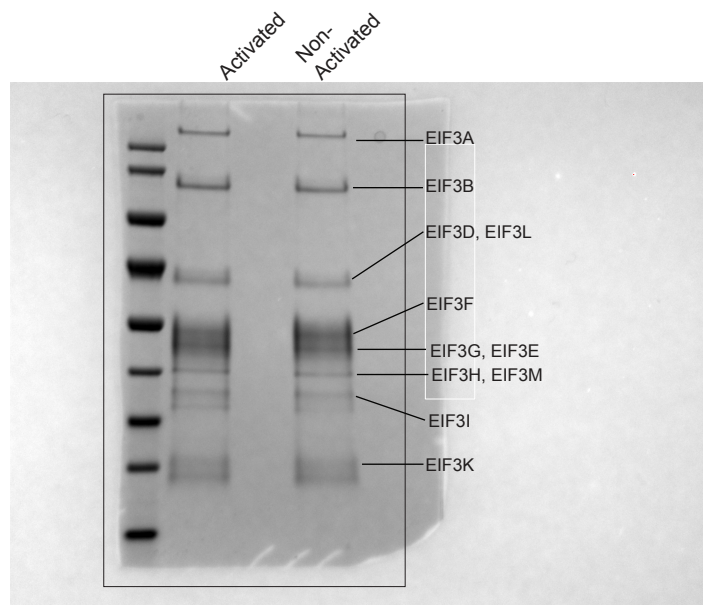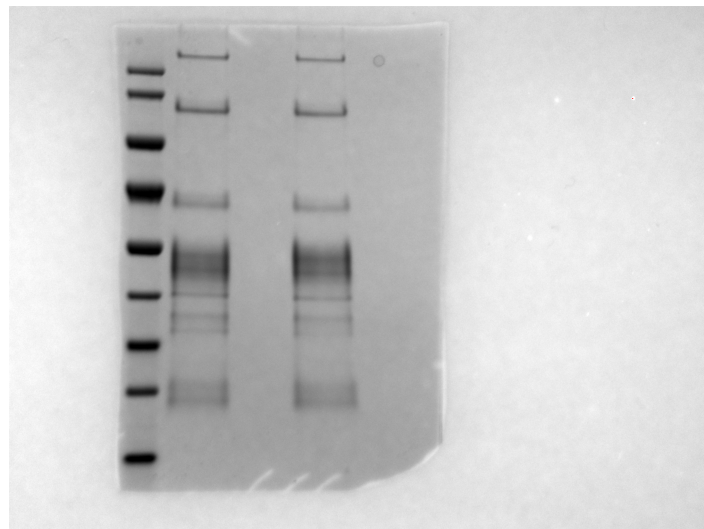

Supplement: Source data 1. — Boxed regions are the regions shown in the figures. [file elife-74272-supp7.zip › DeSilva_Source_Data_Gels-revised/Figure_1_S1A.pdf]

Figure\_6\_S1

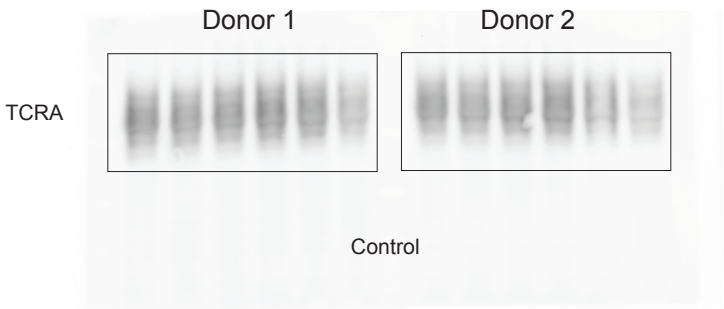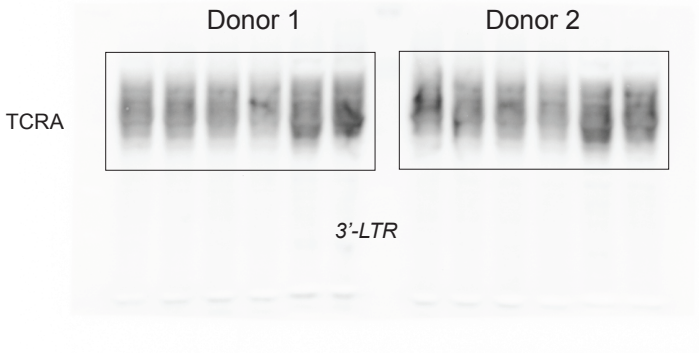

TCRA

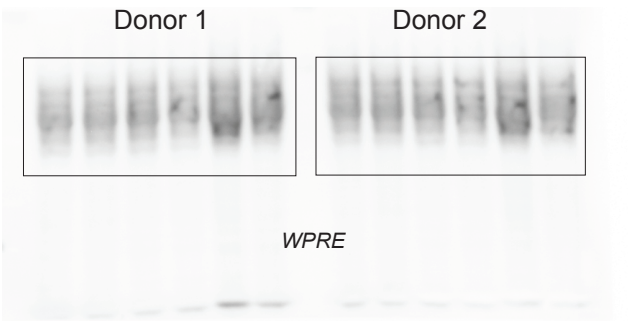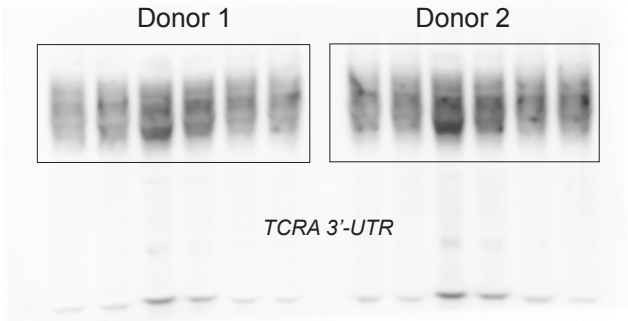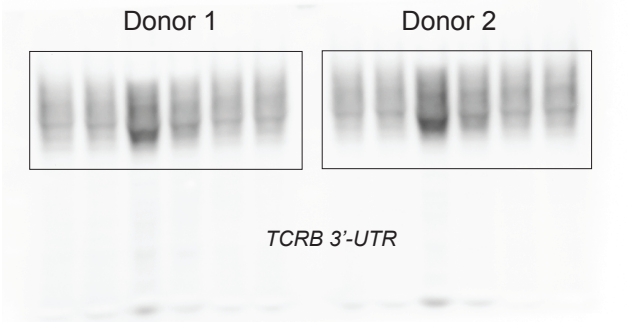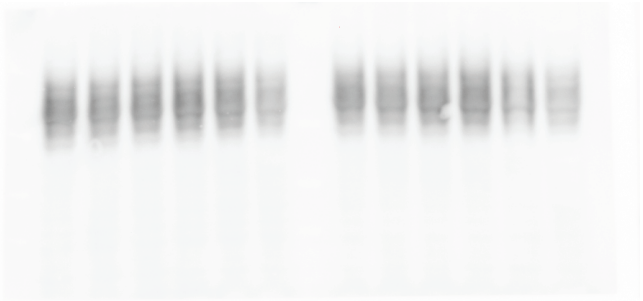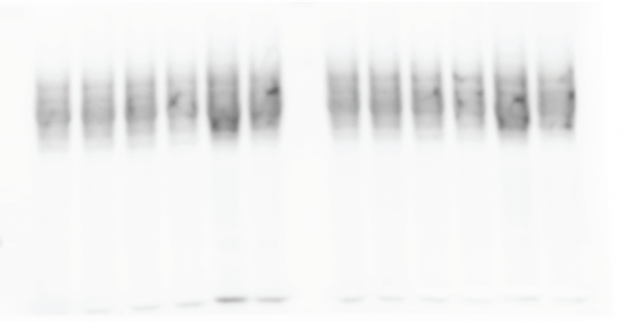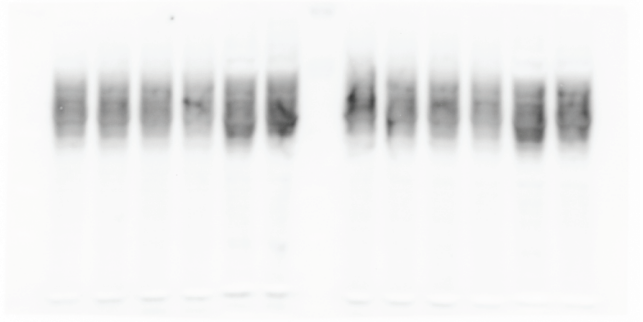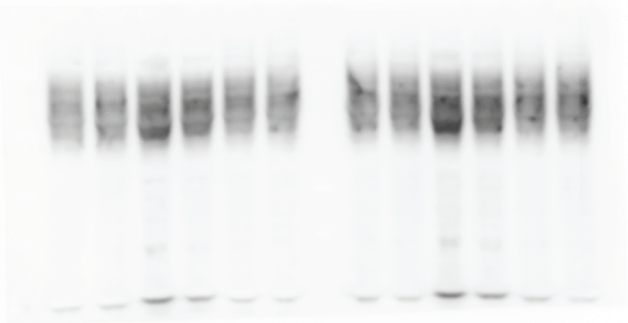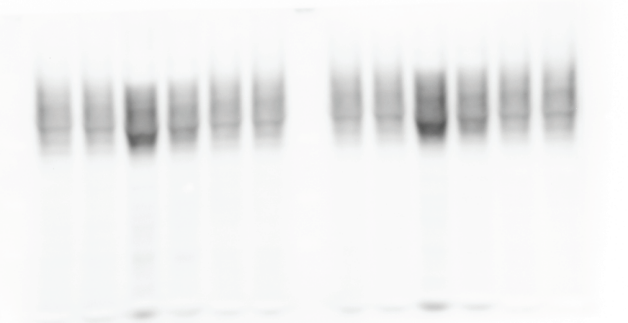

Supplement: Source data 1. — Boxed regions are the regions shown in the figures. [file elife-74272-supp7.zip › DeSilva_Source_Data_Gels-revised/Figure_6_S1.pdf]

Figure\_4\_S1

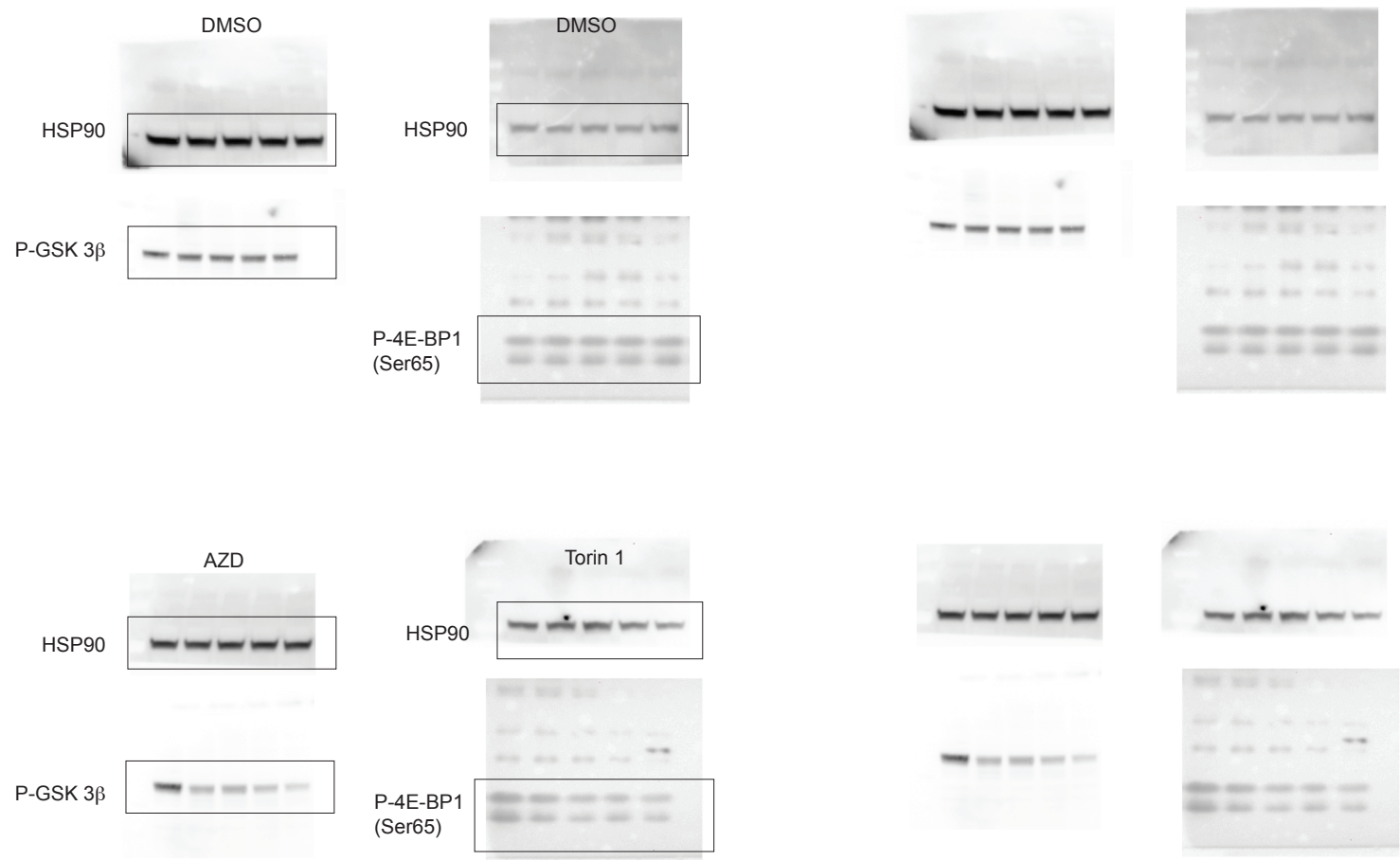

Supplement: Source data 1. — Boxed regions are the regions shown in the figures. [file elife-74272-supp7.zip › DeSilva_Source_Data_Gels-revised/Figure_4_S1.pdf]

Figure\_5\_S1

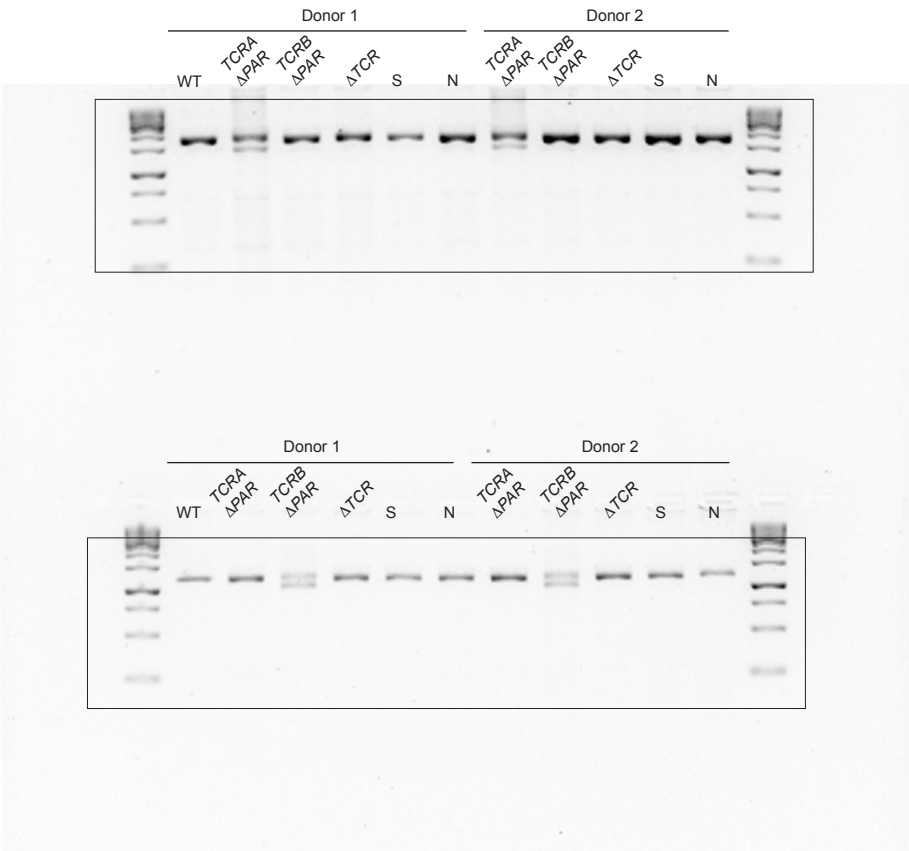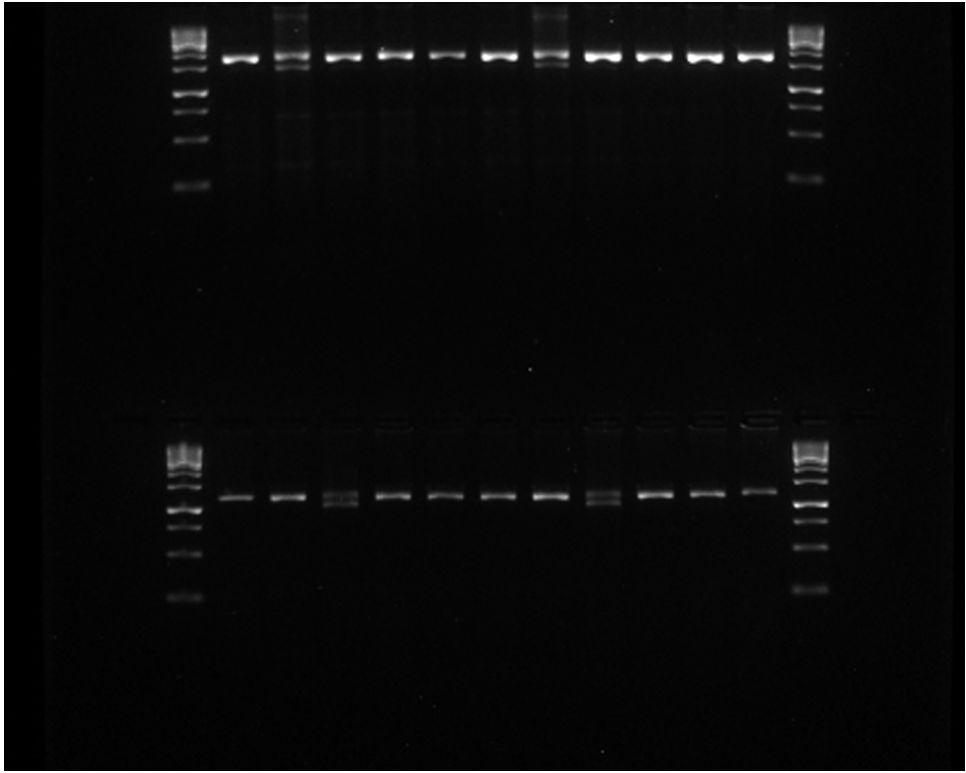

Supplement: Source data 1. — Boxed regions are the regions shown in the figures. [file elife-74272-supp7.zip › DeSilva_Source_Data_Gels-revised/Figure_5_S1B.pdf]

Figure\_5\_S3

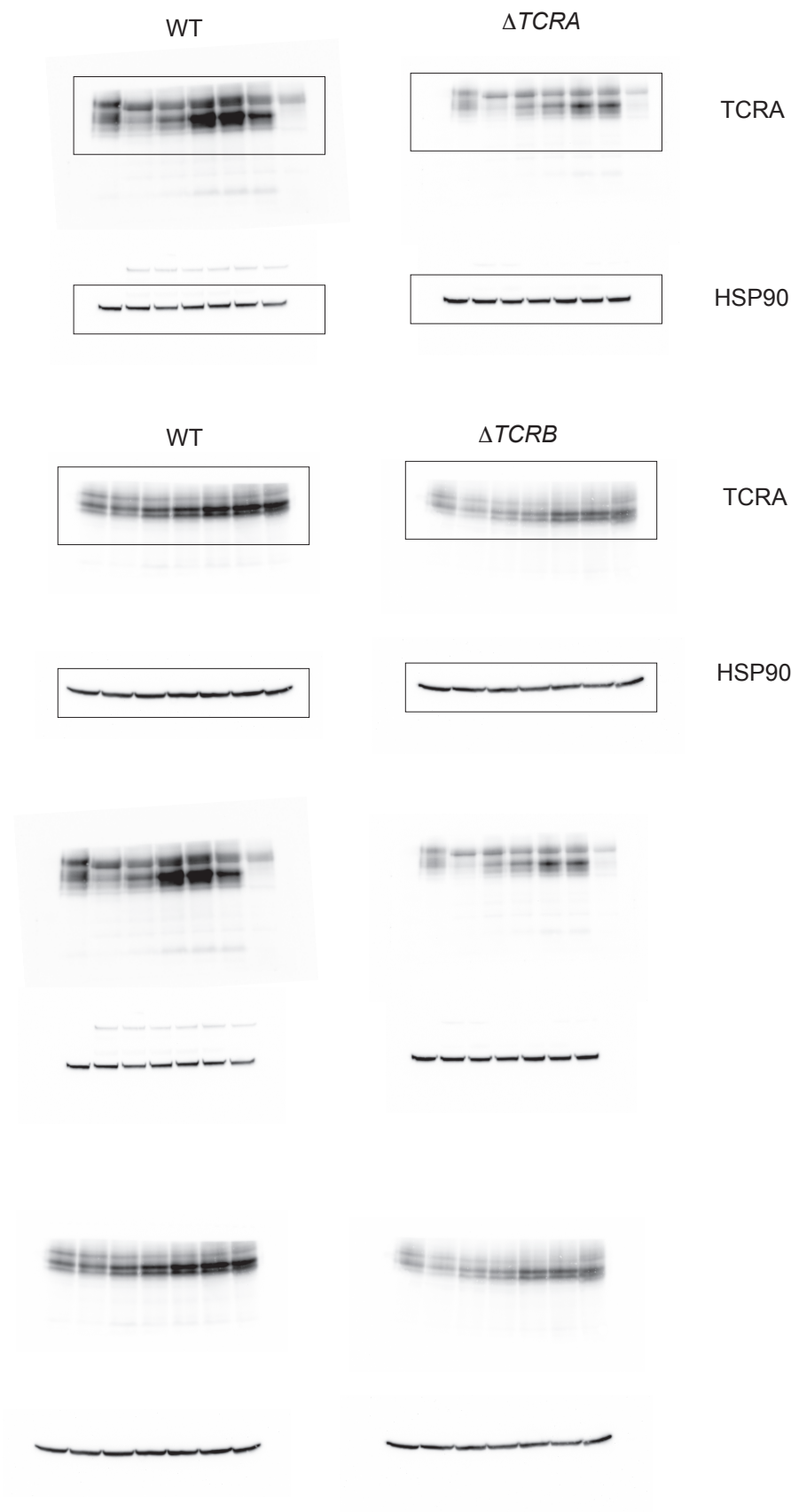

Supplement: Source data 1. — Boxed regions are the regions shown in the figures. [file elife-74272-supp7.zip › DeSilva_Source_Data_Gels-revised/Figure_5_S5.pdf]

Figure 1\_S1

EIF3B

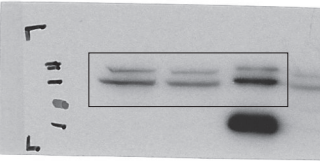

EIF3B

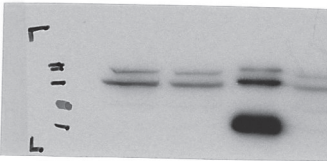

EIF3A

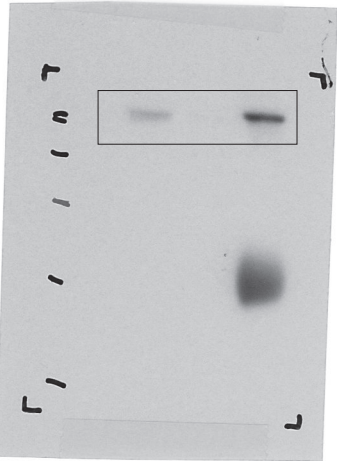

EIF3A

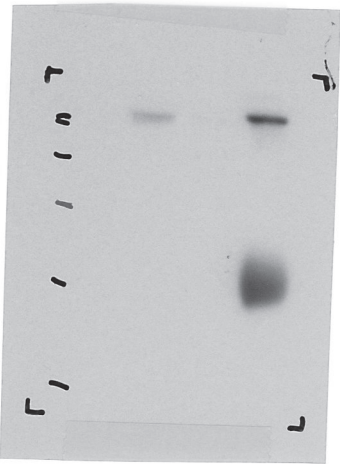

EIF3C

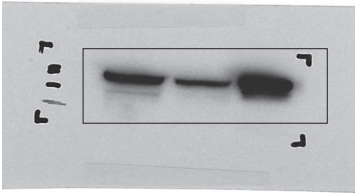

EIF3C

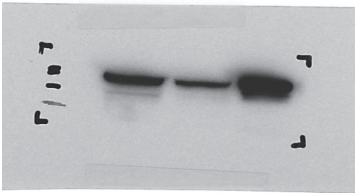

EIF3D

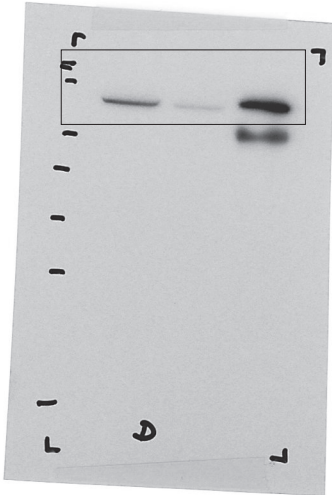

EIF3D

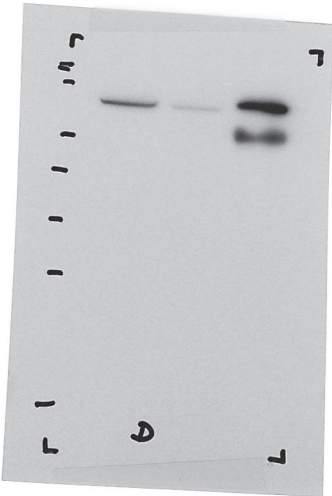

EIF3E

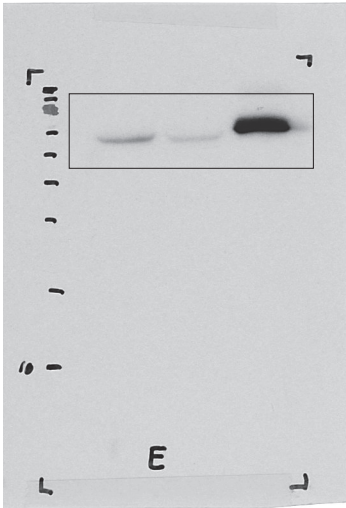

EIF3E

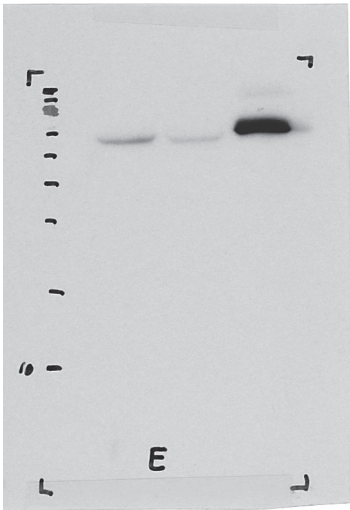

EIF3F

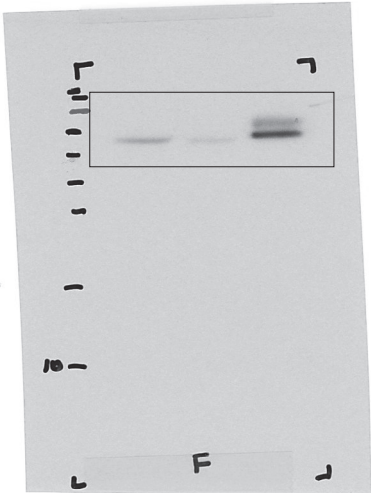

EIF3F

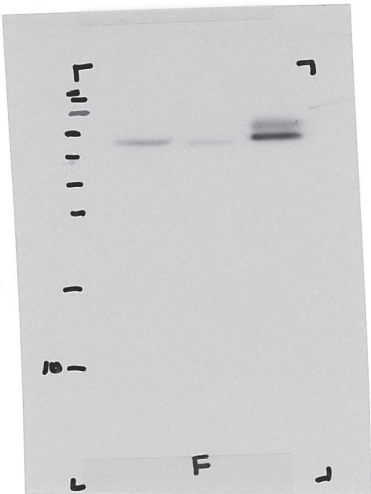

EIF3G

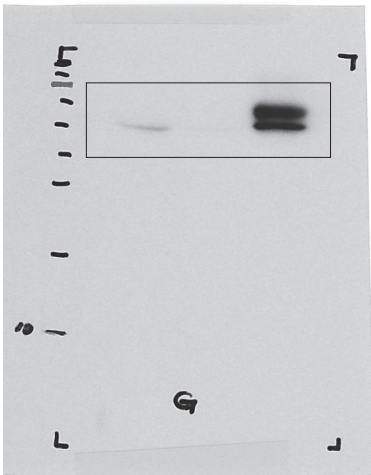

EIF3G

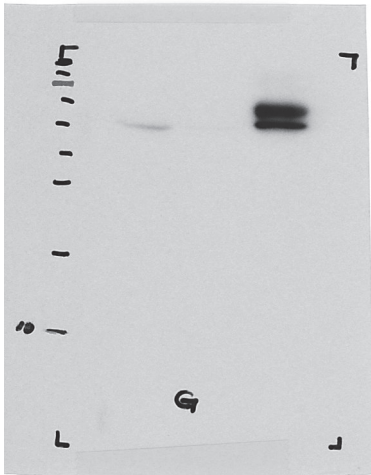

Supplement: Source data 1. — Boxed regions are the regions shown in the figures. [file elife-74272-supp7.zip › DeSilva_Source_Data_Gels-revised/Figure1_S1_page1.pdf]

Figure 1\_S1

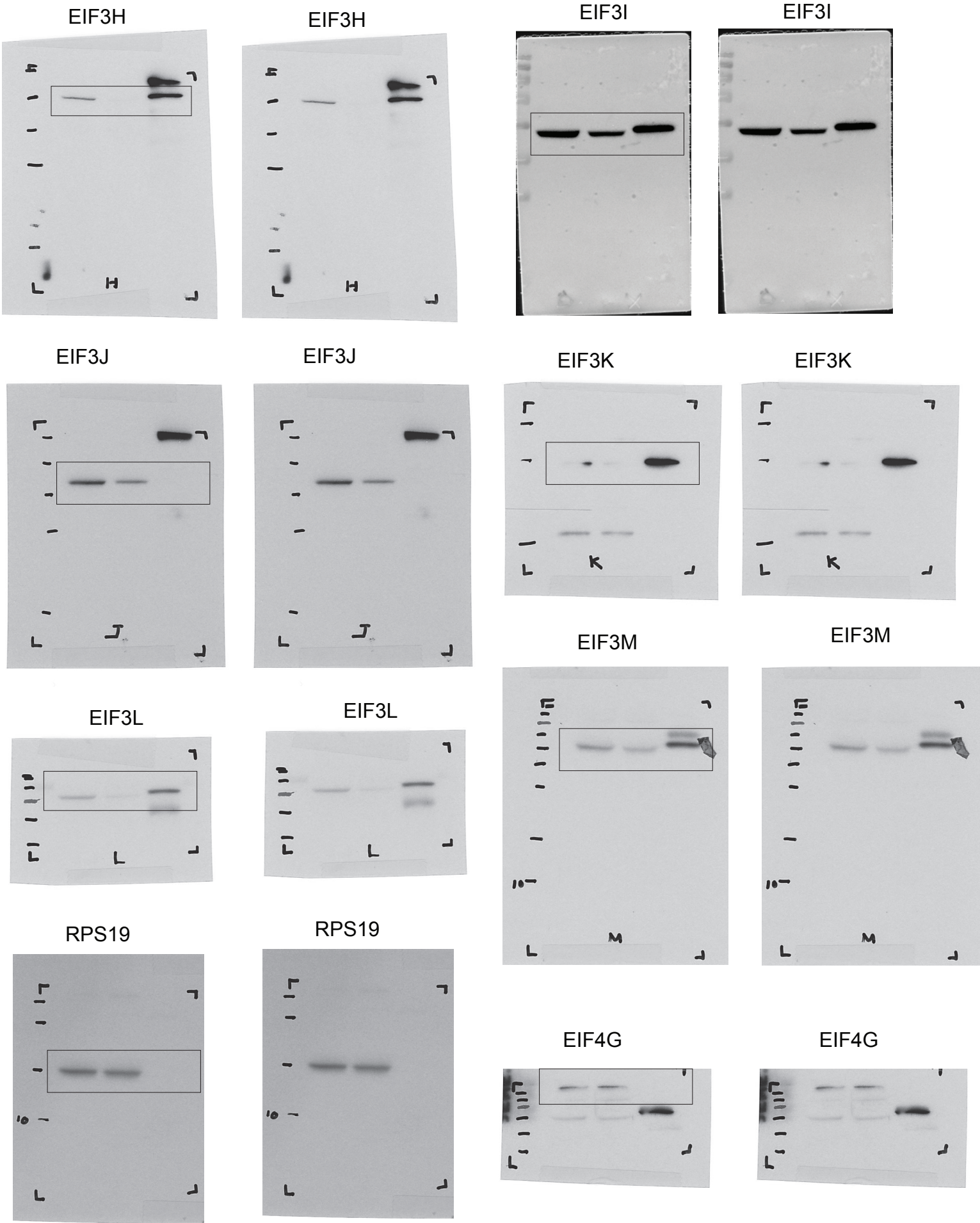

Supplement: Source data 1. — Boxed regions are the regions shown in the figures. [file elife-74272-supp7.zip › DeSilva_Source_Data_Gels-revised/Figure1_S1_page2.pdf]
